# Optimizing Upstream Health in the Community-Oriented Integrative Domain

**DOI:** 10.1089/heq.2018.0041

**Published:** 2018-10-24

**Authors:** Christopher M. Williams, Jehan El-Bayoumi

**Affiliations:** ^1^ Milken Institute School of Public Health, George Washington University, Washington, District of Columbia.; ^2^ The Rodham Institute, School of Medicine and Health Sciences, George Washington University, Washington, District of Columbia.

**Keywords:** academic community partnership, community engagement, health care domains, integrative domain, social determinants of health

## Abstract

Three domains define major challenges facing U.S. health care. Although engagement in the clinical and service domains directly impacts health, integrative engagement operates at the community level to address social determinants of health. Relying on examples from the Rodham Institute, an academically-based community organization, the authors describe principles and practices for promoting community self-efficacy and capacity-building to improve health. These include effective listening, allowing the self-identified needs of the community to drive engagement, and facilitating physician learning involving community education. If social determinants are to be effectively addressed, such approaches provide valuable insight.

Three domains define major challenges facing U.S. health care (Fig. 1). Challenges oriented to clinical medicine involve better care at lower costs within the formal health care system. Whether fee-for-service or value-based care, this domain is characterized by reimbursement for care delivered by specialized health professionals. A service orientation emphasizes an increase in goods and services outside of a hospital or physician payment model, yet seeks to impact health or health-related behaviors. Examples include a two-year grant to reduce obesity prevalence or establishment of a farmer's market in a food desert. Engagement in this domain can be susceptible to shifting priorities and funding shortages. An integrative approach is distinct in that health-related goals operate at a community level to address social determinants of health (SDOH). Equally important is that prioritization of needs and program planning are interwoven with community capacity-building and self-determination. Integrative engagement relies on direct community participation to develop the clinical equivalent of a history and physical, differential diagnosis and treatment plan. Action at this level values principles of reciprocity and ongoing interpersonal communication to advance shared goals of community health.

**FIG. 1.**
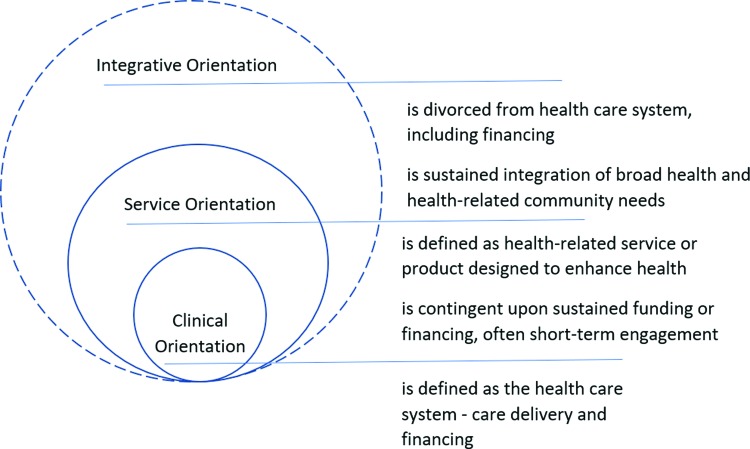
Three domains of health care challenges.

Since its inception in 2013, the Rodham Institute (RI) has emphasized engagement within the integrative domain. A nonprofit within the George Washington University (GWU) School of Medicine and Health Sciences, its priority areas—community collaboration, health profession workforce pipeline, and SDOH education—seek sustainable programming through strong partnerships to facilitate goal setting to address upstream SDOH. The aims intentionally eschew direct patient care and development of downstream health-related goods or services. Rather, the primary role of RI is to listen, to collaborate, and to convene on matters of health, broadly understood, by working in support of community strengthening through collaboration.

Effective listening furthers several principles of community engagement: to acquire knowledge about the community's history and culture, to promote trust building and relationship building, and to respect self-determination. RI pursues data gathering and stakeholder engagement before developing programming aimed at alleviating health inequity in Washington, DC. Early in its history, it commissioned a cross-sectional qualitative research study with key informants such as policy makers, residents, activists, and funders. Mental illness emerged as the most important health priority, followed by stress-related disorders, obesity, and access to quality health care. That work, along with feedback from its annual summits, informed the future direction of RI. For example, seed grants have funded projects to develop a youth mental health first aid kit and toolkits for parents and families of lesbian, gay, bisexual, transgender, and questioning (LGBTQ) youth. Community collaboration also extended to consultative support to a local church's application to establish on-site mental health services. By leveraging its extensive network, RI identified service providers willing to staff the clinic. Support for self-determination is also reflected in free grant-writing workshops for community-based organizations and development of a leadership certificate program. Both initiatives originated in response to expressed needs from community leaders.

Recruitment and retention of a diverse health care workforce is widely recognized as an ongoing challenge that requires strengthening along the educational continuum.^1^ In its 2004 landmark report calling for renewed efforts, the IOM cited benefits of racial and ethnic workforce diversity related to increased access to health care and patient satisfaction among communities of color.^2^ RI had recognized an opportunity to coordinate activity in various educational and nonprofit sectors focused on health career preparedness among youth, so it established *Pathways for All to Health Careers* (PATH-C)—a DC-based consortium to increase the number of under-represented groups in the health care workforce, with emphasis on middle-school-aged African American boys. PATH-C offers STEM-H (Science, Technology, Engineering, Mathematics, and Health Care) career mentorship, didactics, and service learning. Similarly, RI's workforce development program—Health Education and Leadership Program (HELP)—seek to diversify the health care workforce pipeline by exposing high school and middle school students, many from Title I schools, to health career alternatives and training. Participants can earn certifications and receive work-based training from industry professionals, university-affiliated clinicians, and community members. PATH-C and HELP's success in attaining a 100% college enrollment rate attenuates known negative effects of low educational achievement on lifetime health. Although a health care career is encouraged, RI considers students' early exposure to medicine beneficial to individual, family, and community health, even if a health-related career is ultimately not pursued.

Many studies have raised the alarm about the health and well-being of health care professionals, including physicians.^3^ Higher rates of suicide and burnout have led to calls (“Quadruple Aim”) that provider wellness is essential to further aims toward better-quality patient care and improved population health at reduced costs (“Triple Aim”). Downstream health cannot be achieved without a supported health care workforce. As such, RI has incorporated wellness in its “Community Health Connects” that have explored topics related to the humanities and outdoor recreation to enhance self-care.

SDOH—economic well-being, education, health and health care, built environment, and social characteristics—reflect the root causes of diseases. Yet, most clinicians do not feel able to address SDOH influences.^4^ RI promotes education with its Teach-In series that explores various SDOH facets, including housing and food insecurity. These events, which take place in the hospital, attract a diverse audience from hospital employees such as nursing staff, physicians, physical and occupational therapists, and hospital administrators to medical, public health, and nursing students. The educational programs pair an academic MD and not-for-profit representative. In addition, RI sponsors an SDOH field trip (“See the City You Serve”) for first-year residents to economically challenged parts of Washington, DC, included in GWU Hospital's catchment area. Members of RI's Education Council, which comprises an interprofessional team of health care professionals and community members, encourage observation and SDOH discussion during bus transit to a community hospital that hosts a panel discussion. Postsurvey results show significant improvement in residents' understanding of patients' neighborhoods, challenges to health care access for low-income patients, and community resources to support health.^5^

As the given examples illustrate, operating in the integrative domain requires direct involvement of the community in educational programming, even those activities directed at clinician education. RI's Continuing Education for Frontline Clinicians pairs an academic MD and a not-for-profit representative to present topics that fulfill recertification requirements and that reflect clinicians' topic interests such as “HIV and women.”

RI relies heavily on resource sharing within GWU and external entities. RI has cultivated an extensive network of community-based organizations and volunteers who are central to all engagements. When RI had sought to meet a community need for developing grant writing skills, it partnered with a DC-based community health center to provide space and relied on GWU sponsorship to cover speaker expenses. Costs to RI for “See the City You Serve” involve chartering several buses, whereas community and interprofessional faculty volunteers offset considerable potential costs. When community leaders had approached RI for help in development of a leadership certificate program, RI networked with GWU faculty to identify an existing curriculum, which is being tailored to local community goals and needs.

Interprofessional collaboration takes on new meaning within the integrative domain. Although the nature of engagement will vary widely depending on the community, the integrative domain seeks to ensure that communities' perspectives and direct engagement remain forefront in efforts to improve community health.
